# A newly identified mutation (c.2029 C > T) in SLC26A4 gene is associated with enlarged vestibular aqueducts in a Chinese family

**DOI:** 10.1186/s12920-022-01200-4

**Published:** 2022-03-06

**Authors:** Ting Wu, Limei Cui, Yakui Mou, Wentao Guo, Dawei Liu, Jingjing Qiu, Cong Xu, Jiamin Zhou, Fengchan Han, Yan Sun

**Affiliations:** 1 Qingdao University, 308 Ningxia Road, Qingdao, 266071 Shandong People’s Republic of China; 2 Department of Otorhinolaryngology Head and Neck Surgery, The Affiliated Yantai Yuhuangding Hospital of Qingdao University, No. 20 East, Yuhuangding Road, Yantai, 264000 Shandong People’s Republic of China; 3 Key Laboratory for Genetic Hearing Disorders in Shandong, Binzhou Medical University, 346 Guanhai Road, Yantai, 264003 Shandong People’s Republic of China; 4 Transformative Otology and Neuroscience Center, Binzhou Medical University, 346 Guanhai Road, Yantai, 264003 Shandong People’s Republic of China

**Keywords:** Hearing loss, SLC26A4, Mutation, c.2168A > G, c.2029C > T, Enlarged vestibular aqueduct

## Abstract

**Background:**

The enlarged vestibular aqueduct (EVA), associated with mutations in the SLC26A4 gene, characterized by non-syndromic hearing loss, is an autosomal recessive disorder. Here, we intended to investigate genetic causes of hearing loss in a Han Chinese man.

**Method:**

First, whole-exome sequencing was performed to identify the gene mutations responsible for hearing loss in the proband. Sanger sequencing was used to verify the candidate mutations detected in the family. Next, we collected blood samples and clinical data from the three-generation pedigree. Finally, SLC26A4 mRNA and protein expression levels were detected by qPCR and western blotting.

**Result:**

The proband suffered from bilateral progressive sensorineural hearing loss with EVA. The sequence analysis of SLC26A4 revealed that the proband and his sister both harbored a compound heterozygous mutation of c.2168A > G/c.2029C > T, inherited from their father and mother respectively. c.2029C > T mutation has not been recorded in the relevant literature previously. Relative mRNA levels of the SLC26A4 gene in individuals carrying a compound heterozygous mutation were significantly lower compared to a heterozygous mutation. SLC26A4 protein levels of 293t cells which transfected with recombinant plasmids [GV219-SLC26A4-mut (c.2029C > T) and GV219-SLC26A4-mut (c.2168A > G/c.2029C > T)] were significantly lower than normal control recombinant plasmids (GV219-SLC26A4-wt).

**Conclusion:**

This study found a novel heterozygous mutation c.2029 (exon17) C > T compound with c.2168 (exon19) A > G in the *SLC26A4* gene in a patient with EVA. The c.2029 (exon17) C > T mutation is proved to be pathogenic. This finding broadens the spectrum of variants in SLC26A4 gene.

**Supplementary Information:**

The online version contains supplementary material available at 10.1186/s12920-022-01200-4.

## Background

Hearing loss is one of the most prevalent sensory dysfunctions, with a significant influence on the quality of life and physical and mental well-being of the affected individuals in some degree [1, 2]. In China, it is estimated that 30,000 babies are born with congenital hearing loss every year [3]. As far as we know, at least 60% of early-onset hearing loss or hypoacusis cases are correlated with a genetic cause [4]. Most hereditary hearing loss is sensorineural (SNHL) and inherited as a simple Mendelian trait. The hereditary SNHL is classified as autosomal recessive (80%), autosomal dominant (15–20%), x-linked (1%) or mitochondrial inheritance (1–5%) [5].

A mutation in the SLC26A4 gene is the second most common contributor to genetic hearing loss after GJB2 mutations [6]. Mutations in the SLC26A4 gene are involved in syndromic deafness featured on congenital sensorineural hearing impairment and goiter (Pendred syndrome, OMIM 274600) [7] as well as in congenital isolated deafness (DFNB4; OMIM 600791), both of which are relevant to an enlarged vestibular aqueduct (EVA) [8]. EVA, a common type of autosomal recessive hearing loss, is the most common radiological deformity of the inner ear relevant to hereditary SNHL [9].

The SLC26A4 gene is located on chromosome 7q22-31, contains 21 exons, and encodes a highly hydrophobic membrane protein named pendrin, which is a member of the SLC26 family of anion transporters [10]. Pendrin is primarily presented in the thyroid and the inner ear [11]. The complete loss of pendrin-induced anion transport is deemed as cause Pendred syndrome, while function-reducing mutations cause DFNB4, goiter being absent or having a late onset [12]. The SLC26A4 gene has an extensive mutation spectrum spreading across all exons and their flanking sequences. Missense mutations account for a large percentage of SLC26A4 mutations, the rest of which is frameshift mutations, splice site mutations, insertions or deletions that create a stop codon. The entire spectrum of known mutations in the SLC26A4 gene can be viewed at https://deafnessvariationdatabase.org/gene/SLC26A4.

In this paper, we identified a compound heterozygous mutation of c.2168A > G/c.2029C > T in SLC26A4 which was associated with EVA. This will prolongate the scope of knowledge of the spectrum of SLC26A4 mutations in the Chinese population, and it is beneficial for us to explore the relationship between phenotype and genotype in the mutations.

## Methods

### Subjects

The study investigated non-syndromic autosomal dominant sensorineural deafness in a Chinese pedigree. This work and study protocol were approved by the Medical Ethics Committee of the Yantai Yu Huang ding Hospital, Qingdao University, Yantai, China. The genealogical proband was a 20-year-old man recruited from the Department of Otolaryngology Head and Neck Surgery of the Yantai Yu Huang ding Hospital in December 2018. Written informed consents were gained from the pedigree. Furthermore, all of the participants consented to the publication of their results on clinical characteristics and genetic data on the premise of privacy protection.

### Audiological and imaging evaluation

A physical examination was conducted in the pedigree, including a CT of the temporal bone, functional thyroid tests and thyroid sonography. A specific medical history was acquired, including instances of family history of hearing loss and consanguineous marriages, history of ototoxic drugs and noise exposure, and years of onset of deafness, age, progression, Comprehensive audiometric evaluations are composed by distortion product otoacoustic emission (DPOAE), auditory brainstem response (ABR), tympanometry, pure tone audiometry (PTA). Normal hearing was classified as PTA ≤ 25 dB HL (Hearing Level), mild hearing loss as 25 < PTA ≤ 40 dB HL, moderate hearing loss as 40 < PTA ≤ 60 dB HL, severe hearing loss as 60 < PTA ≤ 80 dB HL, and profound hearing loss as PTA > 80 dB HL [13]. A patient was diagnosed with EVA if the midpoint between the common crus and the external aperture was > 1.5 mm by temporal bone CT [14] or MRI.

### SLC26A4 mutation screening

#### Whole-exome sequencing (WES)

The genome variants of the proband (III-1) were identified by whole-exome sequencing by Yin Feng Biotechnology Company (Jinan, China). Potential pathogenic variants were identified in the DNA of the other subjects (I-1, I-4, II-1, II-2, II-3, II-4, II-5, II-6, and III-2). DNA was extracted from the sample, and the main DNA quality testing procedures were as follows: (1) the DNA concentration was tested by Nanodrop lift (thermo scientific, CA, USA);(2) the purity was confirmed using a Nanophotometer (IMPLEN, CA, USA); and (3) the integrity was detected by 1% agarose gel electrophoresis.

The libraries were tested for: (1) exon enrichment using a Sure Select XT Target Enrichment System (G7530–90000); (2) for size distribution using an Agilent 2100 Bioanalyzer system; and (3) for concentration using a Bio-Rad CFX 96 quantitative fluorescence PCR instrument and Bio-Rad KIT iQ SYBR GRN. The samples were then sequenced on a NovaSeq 6000 platform. Variant analysis software (GATK) was used to extract the potential sites of SNP and InDel in the whole genome and compared to the reference database Human_GRCh38_dbSNP141.

#### Sanger sequencing

SLC26A4 gene variants identified by WES in the pedigree were confirmed by Sanger sequencing. Genomic DNA of all family members was prepared from 3 mL of peripheral blood with a QIAGEN Universal DNA Purification kit. The primer pairs are present in Table 1. PCR was carried on according to these steps: an initial denaturation at 96 °C for 1 min, 96 °C for 10 s (denaturation), 50 °C for 5 s (annealing), 60 °C for 4 min (extension) and a final 25 cycles. In the end, the PCR products were sequenced with an ABI 3730 Sequencer (Applied Biosystems).

**Table 1:** The primer pairs used in the present study

Gene	Forward sequence (5′–3′)	Reverse sequece (5′–3′)
SLC26A4(c.2029)	GCTGAGGTGAAACCCATCCT	AGCCCATGTATTTGCCCTGT
SLC26A4(c.2168)	CCACAAGGTTGACTACGACCA	CCAGATGCGCTACTGTTGTG
SLC26A4 qPCR	GTGGGATCTGTTGTTCTGAGCAT	GGCACTGGCAATCAGGACTC
Actin qPCR	CACCATTGGCAATGAGCGGTTC	AGGTCTTTGCGGATGTCCACGT

### Detection of relative mRNA expression levels of SLC26A4

Peripheral blood samples were collected from all family members. RNA was isolated using Trizol reagent and reverse transcripted into cDNA according to the manufactures’ protocols (Accurate biology, Hunan, China). Relative mRNA levels of the SLC26A4 target gene were detected by quantitative PCR performed on an Applied Biosystems StepOnePlus system (Foster City, CA). Primer pairs were showed in Table 1. Data were collected and the 2^−ΔΔCt^ method was used to determine the relative mRNA expression level.

### Detection of protein levels of SLC26A4

Three groups of recombinant overexpression plasmids containing a SLC26A4 single mutation site (GV219-SLC26A4-mut (c.2029C > T)), double mutation sites (GV219-SLC26A4-mut (c.2168A > G/c.2029C > T)) and wild-type (GV219-SLC26A4-wt) fragments were constructed by Shanghai Genechem Co., Ltd. The recombinant vectors were transiently transfected into 293 T cells for overexpression. Transfected cells were cultured for 48 h and then collected for western blotting analysis. The SLC26A4 antibody (NBP1-60106) is from Novus Biologicals.

## Results

### Family investigation and clinical evaluation

**Fig. 1:**
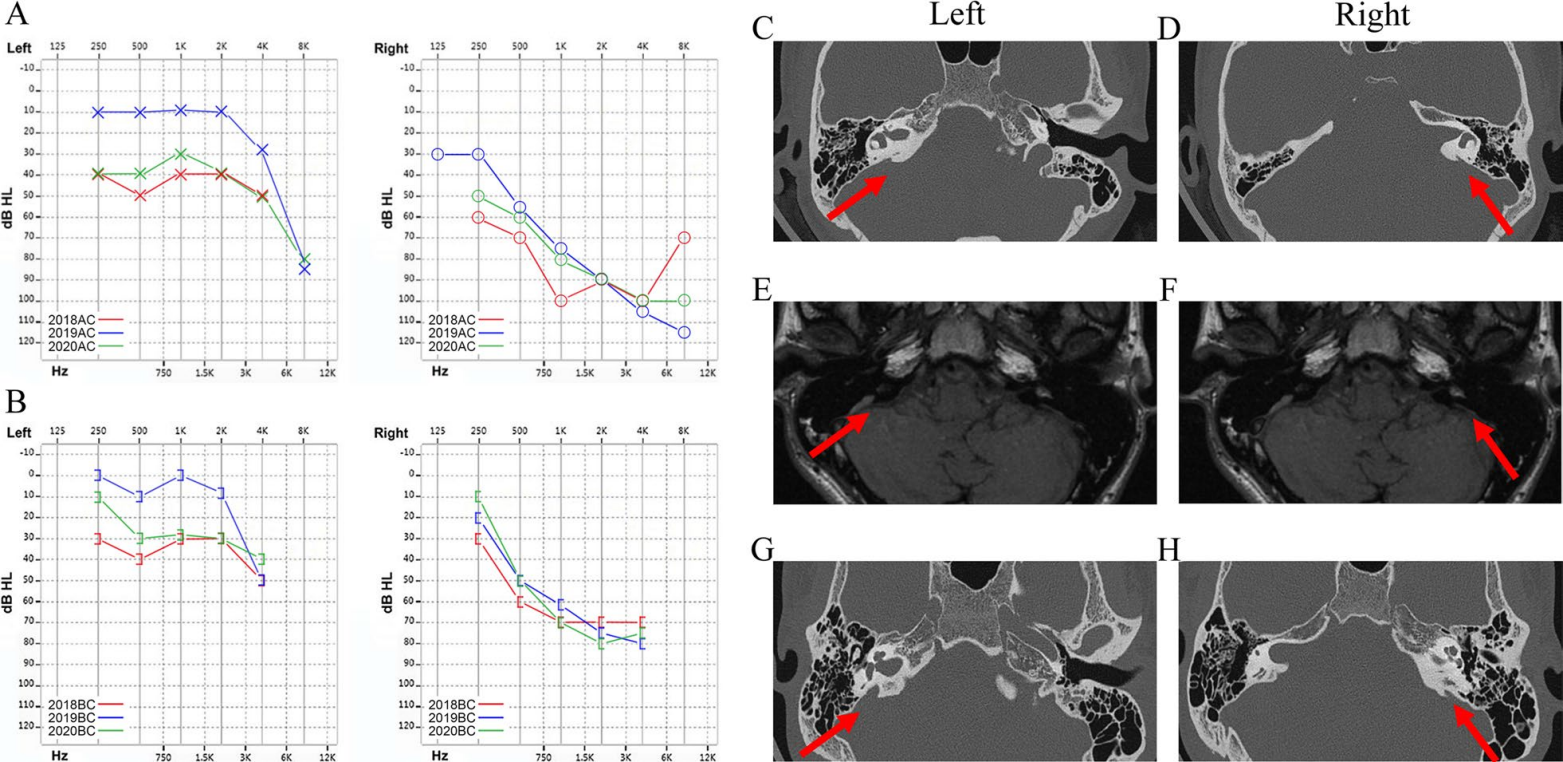
Results of the audiogram function test and imagological examination. **a** PTA showed the change of air conduction hearing in both ears of the proband in 2018, 2019 and 2020. **b** PTA showed the change of bone conduction hearing in both ears of the proband in 2018, 2019 and 2020. **c** and **d** CT scans showed the EVA in both ears (red arrows) in III-1. **e** and **f** MRI scans showed the lymphatic enlargement in both ears (red arrows) in III-1. **g** and **h** CT scans showed the EVA in both ears (red arrows) in III-2. *dB* decibels, *HL* hearing level, *CT* computed tomography, *MRI* magnetic resonance imaging

Here we identified a three-generation Han Chinese family with 10 members. The proband (III-1) had normal hearing at birth. However, when he was 18, he developed sensorineural deafness in the right ear and mild hearing loss in the left ear after a bad cold. The results presented in Fig. 1a and b showed a progressive aggravation of hearing loss at 19 and 20 years old. On tympanogram, the proband was classed as type A; CT scans of the inner ear showed EVA bilaterally without cochlea malformations (Fig. 1c, d) (Additional file 1: Supplementary figure 1.a, b ); and MRI showed lymphatic enlargement and no vestibular endolymphatic hydrops (Fig. 1e, f) (Additional file 1: Supplementary figure 2.a, b). Thyroid goiter was excluded based on normal thyroid function tests and ultrasonogram. Overall, the proband (III-1) was correlated with non-syndromic hearing loss with EVA. Another member of this family (III-2) also had EVA and mild hearing loss on PTA with no clinical symptoms (Fig. 1g, h) (Additional file 1: Supplementary figure 3.a, b). None of the family members had a history of ototoxic drug exposure. The clinical phenotype presentations of the pedigree are shown in Table 2.

**Table 2:** Clinical characteristics of the family individuals

Subject	Age (years)	Sex	Pure tone test	Degree of hearing loss	DPOAE	Binaural ABR threshold (dB HL)	Tympanometry
I-1	72	Male	4000–8000 HZ frequency sensorineural deafness in left ear	Mild on left side	1000 HZ, 1500 HZ frequency extraction in normal range in left ear	40 in right ear	Binaura “A” curve
					700–1500 HZ frequency extraction in normal range in right ear	50 in left ear	
I-4	71	Female	Binaural high-frequency sensorineural deafness	Mild on both side	Binaural full frequency extraction a bin normal range	50 in right ear	Binaura “A” curve
						60 in left ear	
II-1	48	Male	Binaural 2000–4000 HZ frequency sensorineural deafness	Mild on both side	1000–8000 HZ frequency extraction in abnormal range in left ear	40 in both ears	Binaura “A” curve
II-2	45	Male	Binaural normal hearing	Normal hearing	1000–2000 HZ frequency extraction in normal range in left ear	40 in right ear	Binaura “A” curve
					1500 HZ frequency extraction in normal range in right ear	50 in left ear	
II-3	45	Female	Low frequency sensorineural deafness in left ear	Mild on left side	Binaural down 1000 HZ frequency Extraction in normal range	40 in both ears	Binaura “A” curve
II-4	41	Female	Binaural high-frequency sensorineural deafness	Mild on both side	Binaural 500–2000 HZ frequency extraction in normal range	60 in both ears	Binaura “A” curve
II-5	35	Female	Binaural normal hearing	Normal hearing	Binaural 6000 HZ 8000 HZ frequency extraction in abnormal range	30 in both ears	Binaura “A” curve
II-6	31	Male	Binaural high-frequency sensorineural deafness	Mild on both side	Binaural down 2000 HZ frequency extraction in normal range	40 in right ear	Binaura “A” curve
						60 in left ear	
III-1	20	Male	Sensorineural deafness in right ear	Severe profound on both sides	Full frequency extraction in abnormal range in right ear	90 in right ear	Binaura “A” curve
			Sensorineural deafness with high-frequency in left ear		1500–3000 HZ frequency extraction in normal range in left ear	50 in left ear	
III-2	16	Female	Binaural low frequency sensorineural deafness	Mild on both side	Binaural full frequency extraction in normal range	20 in both ears	Binaura “A” curve

*DPOAE* distortion product otoacoustic emission, *ABR* auditory brainstem response, *dB* decibels, *HL* hearing level

### Identification of SLC26A4 gene mutations

**Fig. 2:**
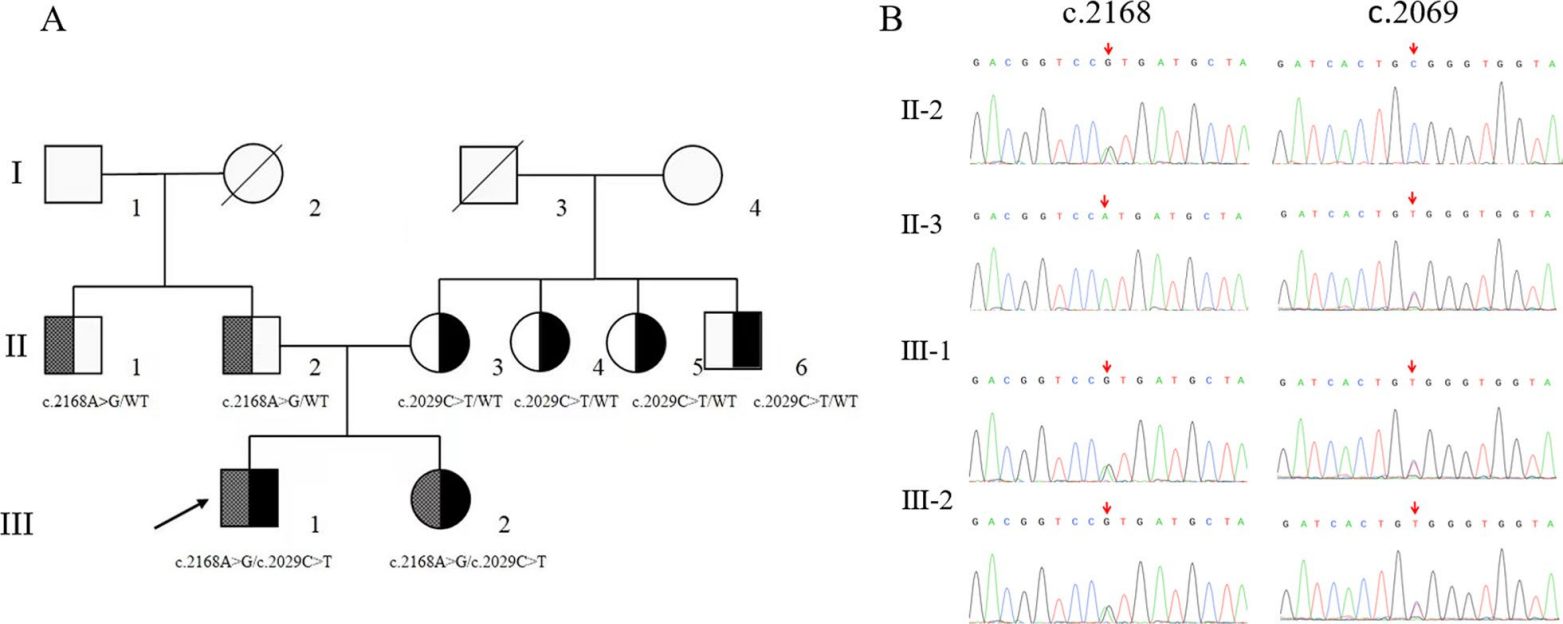
Pedigree map and partial sequence chromatograms of SLC26A4 mutations in the three-generation pedigree. Squares denote males; circles denote females; black denotes c.2029C > T; and gray denotes c.2168A > G. The proband is identified by an arrow

**Fig. 3:**
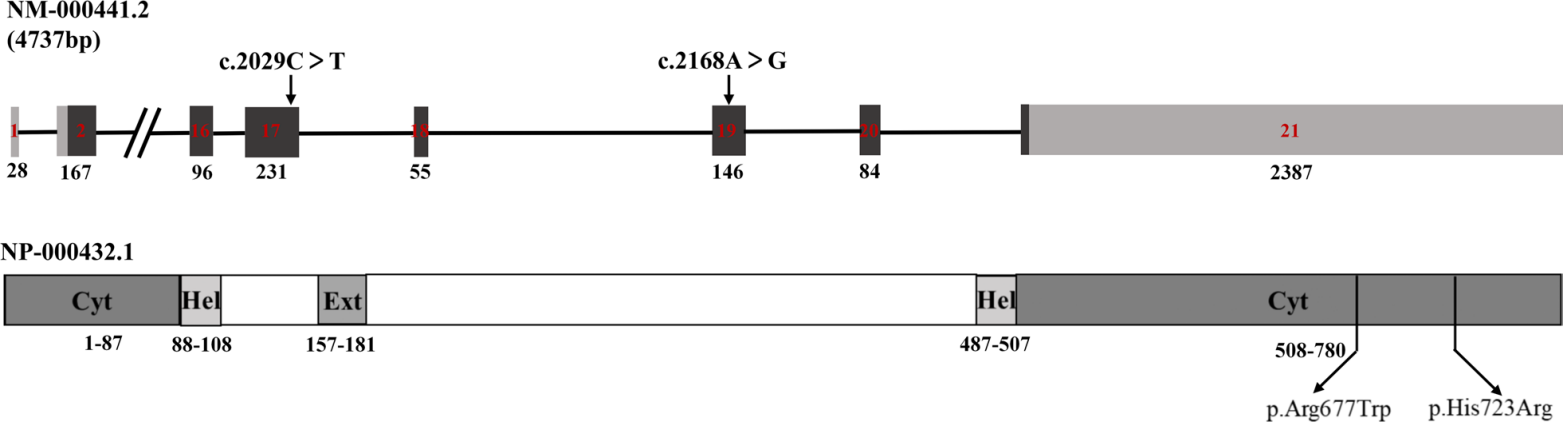
The new missense mutation c.2029C > T in exon 17 led to a substitution of Trp with Arg at position 677, the c.2168 (exon19) A > G mutation led to a substitution of Arg with His at position 723 (black arrow)

A compound heterozygous mutation of SLC26A4 (c.2168A > G/c.2029C > T) was detected in proband III-1 by WES. The results of Sanger sequencing showed that both III-1 and III-2 carried the c.2029C > T and c.2168A > G mutations, however I-4, II-3, II-4, II-5 and II-6 were heterozygous carriers of the c.2029C > T mutation. I-1, II-1 and II-2 were heterozygous carriers of the c.2168A > G mutation. Of note, we were able to determine details of the mutations and the pedigree map. Genotypes of the family members are listed in Fig. 2. The c.2029 (exon17) C > T mutation led to a substitution of Trp with Arg at position 677, the c.2168 (exon19) A > G mutation led to a substitution of Arg with His at position 723 (Fig. 3). The important information of both variants was displayed in Table 3.

**Table 3:** The variant information of SLC26A4 c.2168A > G and c.2029C > T

Nucleotide change	Amino acid change	chromosomal location	rs-numbers	Population frequency (GnomAD) (‰)
c.2168 A > G	p. His723Arg	Chr7:107350577	rs121908362	0.113
c.2029 C > T	p. Arg677Trp	Chr7:107342497	rs397516426	0.0460

### Bioinformatics analysis

**Fig. 4:**
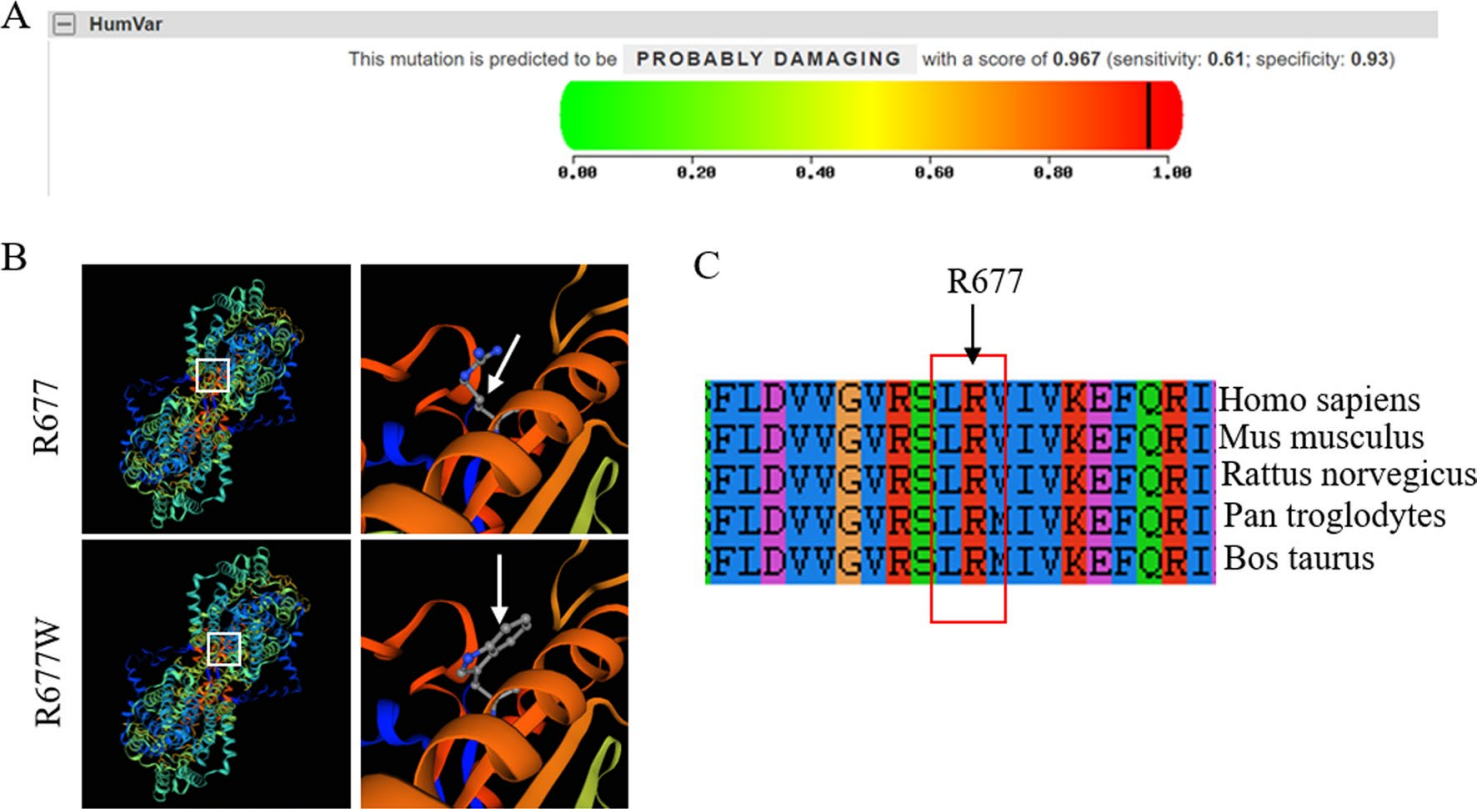
Bioinformatics evaluation of the impact of SLC26A4 R677W mutation. **a** PolyPhen 2 prediction of the probably damaging caused by c.2029 C > T mutation. **b** the 3D protein structures in specific R677 position of wildtype and mutant SLC26A4 protein, the white arrows indicate R677 position. **c** Protein alignment showed conservation of SLC26A4 residues across 5 species in R677 position

The SLC26A4 c.2168 A > G variant has been reported to be pathogenic previously [20, 21], but c.2029 C > T is a novel missense mutation, so far whether the mutation is pathogenic was unknown. In this case, we further dissects the pathogenicity of the new SLC26A4 c.2029C > T, p.Trp677Arg mutation by using several bioinformatics prediction software. PolyPhen-2 (version 2, http://genetics.bwh.harvard.edu/pph2/) was used to predict function of the peptide and the possible impact of an amino acid substitution on the structure. It is specifically designed to predict missense mutations and mainly performs function prediction on SNP and point mutation. It suggested that the SLC26A4 c.2029C > T mutation is probably destructive, with a score of 0.967 (sensitivity, 0.61; specificity, 0.93) (Fig. 4a). In addition, SWISS-MODEL software was used to generated the 3D protein structures in specific R677 position of wildtype and mutant SLC26A4 protein, which were displayed in Fig. 4b. We also use DNAstar Megalign software to analyze the residue conservation of Trp at position 677. The result of multiple protein sequence alignment indicated that the Trp at position 677 of SLC26A4 is evolutionally highly conserved among various species (Fig. 4c). All these bioinformatics analyses strongly suggested that SLC26A4 c.2029C > T is a pathogenic mutation.

### SLC26A4 mRNA and protein expression

**Fig. 5:**
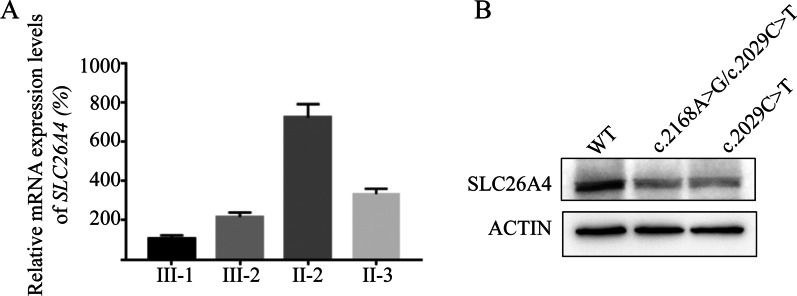
Identified gene and protein expression levels of SCL26A4 with different genotypes by qPCR and western blotting. **a** Relative mRNA expression levels of SLC26A4 gene in PBMCs of different individuals. **b** Relative protein expression levels of SLC26A4 in 293t cells transfected with different recombinant plasmids

To further investigate the impact of compound heterozygous mutation c.2168A > G and c.2029C > T on SLC26A4*,* we analyzed SLC26A4 mRNA expression levels in some individuals of pedigree by qPCR. The results showed that SLC26A4 mRNA levels in III-1 and III-2 with a compound heterozygous mutation (c.2168A > G/c.2029C > T) were significantly lower compared to II-2 and II-3, especially lower than II-2, which only carried a heterozygous c.2168A > G mutation (Fig. 5a). The SLC26A4 protein level in 293 T cells transfected by recombinant plasmids (GV219-SLC26A4-mut (c.2029C > T) and GV219-SLC26A4-mut (c.2168A > G/c.2029C > T) was significantly lower than the wildtype control recombinant plasmids (GV219-SLC26A4-wt, Fig. 5b) (Additional file 1: Supplementary figure 4.a, b).

## Discussion

SLC26A4 mutation was the main cause of EVA, and no other mutations associated with these clinical phenotypes have been found. The incidence of SLC26A4 gene mutations is 20.35%, ranking second only to GJB2 mutations (25.65%) [15]. Some studies believe that the phenotype of EVA has a strong correlation with number of mutant alleles of SLC26A4 in Western populations [16]. Wang et al. [17] investigated 95 Chinese simplex families correlated with EVA maldevelopment, available date suggested that 88.4% of patients carried biallelic variants, monoallelic mutation account for 9.5%, while the other 2.1% did not have a mutant SLC26A4 allele.

We summarized the missense mutations located in exon 19 of the SLC26A4 gene accompanied with pathogenic clinical phenotype in Table 4 based on ClinVar database. We found that the reported SLC26A4 mutations were mainly located between positions 508–708, with clinical manifestations of Pendred syndrome or sensorineural hearing loss. In this study, we discovered that the proband carried a compound heterozygous mutation (c.2168A > G/c.2029C > T) in the SLC26A4 gene and his clinical presentation was profound high frequency hearing loss.

**Table 4:** The missense mutation of exon 19 of the SLC26A4 gene with clinical phenotype as pathogenicity

Nucleotide change	Protein change	Variant classification	Phenotype	Protein position	Consequence
c.2141G > A	p.Arg714Lys	Pathogenic	Hearing loss	714	missense_variant
c.2145G > T	p.Lys715Asn	Pathogenic	Hearing loss	715	missense_variant
c.2153 T > C	p.Phe718Ser	Pathogenic	Enlarged vestibular aqueduct	718	missense_variant
c.2162C > T	p.Thr721Met	Pathogenic	Deafness, non-syndromic, autosomal recessive	721	missense_variant
c.2167C > G	p.His723Asp	Pathogenic	Deafness, non-syndromic, autosomal recessive	723	missense_variant
c.2167C > T	p.His723Tyr	Pathogenic	Enlarged vestibular aqueduct	723	missense_variant
c.2168A > G	p.His723Arg	Pathogenic	Pendred syndrome	723	missense_variant
c.2170G > A	p.Asp724Asn	Pathogenic	Pendred syndrome	724	missense_variant
c.2171A > G	p.Asp724Gly	Pathogenic	Deafness, non-syndromic, autosomal recessive	724	missense_variant
c.2173G > C	p.Ala725Pro	Pathogenic	Pendred syndrome/DFNB4	725	missense_variant
c.2179C > T	p.Leu727Phe	Pathogenic	Enlarged vestibular aqueduct	727	missense_variant
c.2182 T > C	p.Tyr728His	Pathogenic	Deafness, non-syndromic, autosomal recessive	728	missense_variant
c.2219G > T	p.Gly740Val	Pathogenic	Enlarged vestibular aqueduct	740	missense_variant

In general, SLC26A4 mutations give rise to hearing loss, which is more pronounced at high frequency. Yoshida et al. [18] reported a patient, had low-frequency SNHL and endolymphatic hydrops, carring SLC26A4 gene c.1105A > G mutation, In acute low-frequency SNHL, mild or more pronounced endolymphatic hydrops was reported in the cochlea in 88% of vestibula and 82% of ears [19]. EVA patients with bi-allelic mutations had more severe deafness, earlier age of onset, and more fluctuating hearing levels compared to patients with no pathogenic mutations. The proband in this study had an asymptotic high-frequency hearing loss, which was consistent with the bi-allelic mutation phenotype.

The c.2168 A > G variant is considered to be pathogenic [20, 21]. What attracted our attention was that the c.2029 (exon17) C > T mutation in SLC26A4 has already previously been deposited in the ClinVar database, but so far it is not clearly whether the mutation is pathogenic. It has been classified as a variant of unknown significance (VUS). The mutation led to a substitution of Trp with Arg at position 677 of the amino acid. While Trp is a neutral polar amino acid, Arg is an alkaline polar amino acid. We suggest that Arg might be crucial for Pendrin function by influencing the Cl^−^/I^−^ transporter or Cl^−^/HCO_3_^−^ exchange at the level of protein expression and function (Additional file 1).

We discovered that mRNA and protein expression of SLC26A4 with a c.2029 (exon17) C > T mutation is comparatively low. We speculated it may be related to the location of c.2029 C > T. The c.2029 C > T missense mutation located closely to the edge of splice site of exon 17. As splicing site is very important for gene transcription, it may be a possible explanation on the lower mRNA/protein expression levels. Using SWISS-MODEL software, we detected that the substitution of Trp with Arg is able to destabilize the protein conformation, however c.2029 C > T mutation has no significant effect on SLC26A4 3D protein structure. We found a PolyPhen-2 prediction score of 0.967 (sensitivity, 0.61; specificity, 0.93). Generally, the closer the score is to 1, the damage is likely to be. Collectively, these results strongly indicated that the c.2029C > T mutation was likely to be deleterious to the protein. Therefore, we can presume that the c. 2029 (exon17) C > T mutation in the SLC26A4 gene may be a specific mutation closely associated with hearing loss in Chinese patients related to EVA.

SLC26A4 mutations do not change the polarity sensitivity of the auditory nerve fibers to electrical stimulation, which mainly lead to inner ear dysplasia and cochlear dysfunction [22]. Wu Ko et al. [23] indicated that cochlear implants should be valid in patients which carried SLC26A4 gene mutations. The proband in this work may receive single-sided cochlear implantation. Patients with same SLC26A4 genotype may have different phenotypes, whether they are from the same family or not. Chen et al. [24] reported two Chinese patients with a compound heterozygous mutation (c.919A > G and c.1548insC) in SLC26A4. Although they came from the same family, they showed different phenotypes. The younger sister of this proband has the same genotype: temporal bone CT showed that she also had EVA and PTA, indicating a slight hearing loss in both ears. Such a patient may suffer fluctuation of hearing loss which may eventually develop into severe or total deafness after external factors such as colds, coughs, sneezes, or trauma to the head. To prevent those situations, she should try to avoid trauma and other predisposing factors in her daily life.

## Conclusions

In summary, using whole-exome sequencing we identified a new compound heterozygous mutation (c.2168A > G/c.2029C > T) in a young Chinese man which will further strengthen the association between SLC26A4 mutation and EVA. We also demonstrated that the novel mutation (c.2029 C > T) is pathogenic. Further functional studies could assist in clarifying the relationship between the pathogenic mechanism and the novel mutation. In addition, there is still much room to explore the precise molecular mechanism of each mutation and how they affect phenotypic expression.

## Supplementary Information

**Additional file 1** . The original data of  imaging examination and Western bloting examination.

## Data Availability

All data supporting the findings of this study are included in this published article. Meanwhile, data supporting the manuscript is available from any of the two corresponding authors on reasonable request.

## Abbreviations

EVA: Enlarged vestibular aqueduct
SNHL: Sensorineural hearing loss
ABR: Auditory brainstem response
PTA: Pure tone audiometry
DPOAE: Distortion product otoacoustic emission
dB: Decibels
HL: Hearing level
CT: Computed tomography
MRI: Magnetic resonance imaging
WES: Whole-exome sequencing
