# Supplementary material for: A newly identified mutation (c.2029 C > T) in SLC26A4 gene is associated with enlarged vestibular aqueducts in a Chinese family
Source: BMC Med Genomics. 2022 Mar 6;15:49. doi: 10.1186/s12920-022-01200-4 (PMC8898487; doi:10.1186/s12920-022-01200-4)

Supplementary figure.1 CT scans showed the EVA in both ears in III-1.(A) the original image of Figure.1C . (B) the original image of Figure.1D.

A B

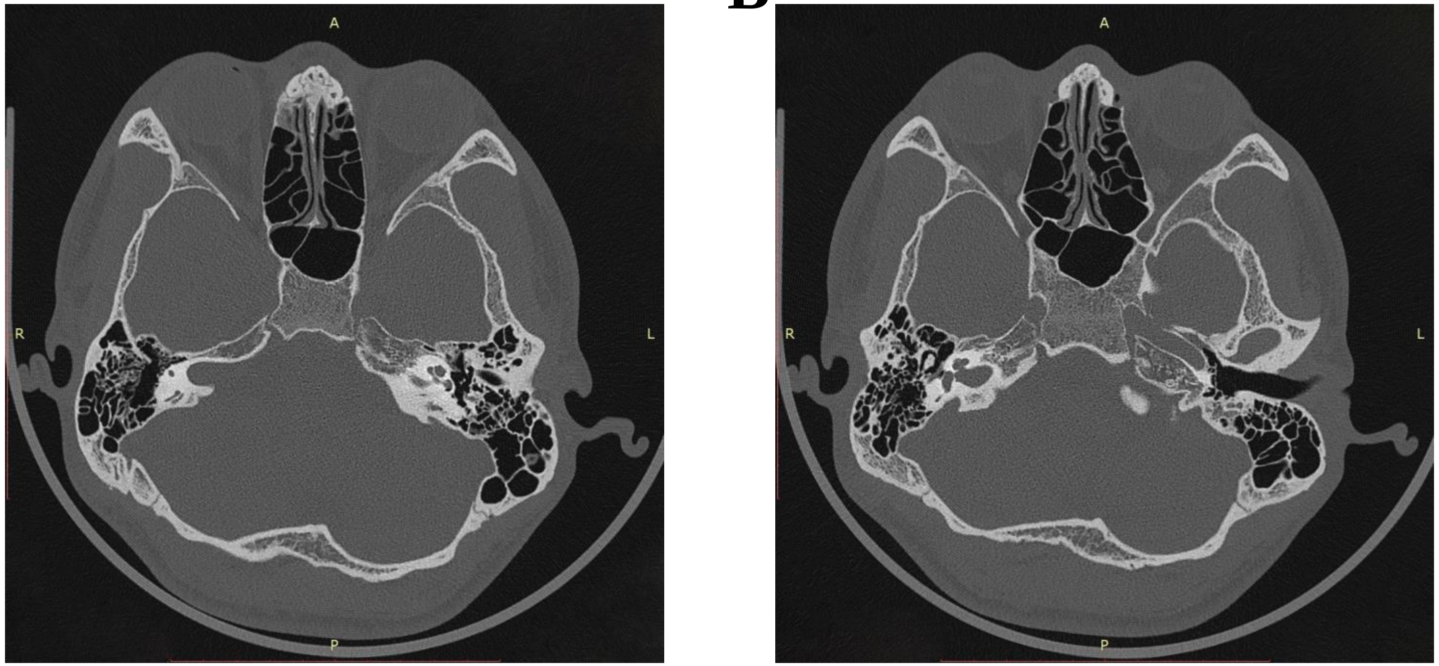

Supplementary figure.2 MRI scans showed the lymphatic enlargement in both ears in III-1.(A) the original image of Figure.1E . (B) the original image of Figure.1F.

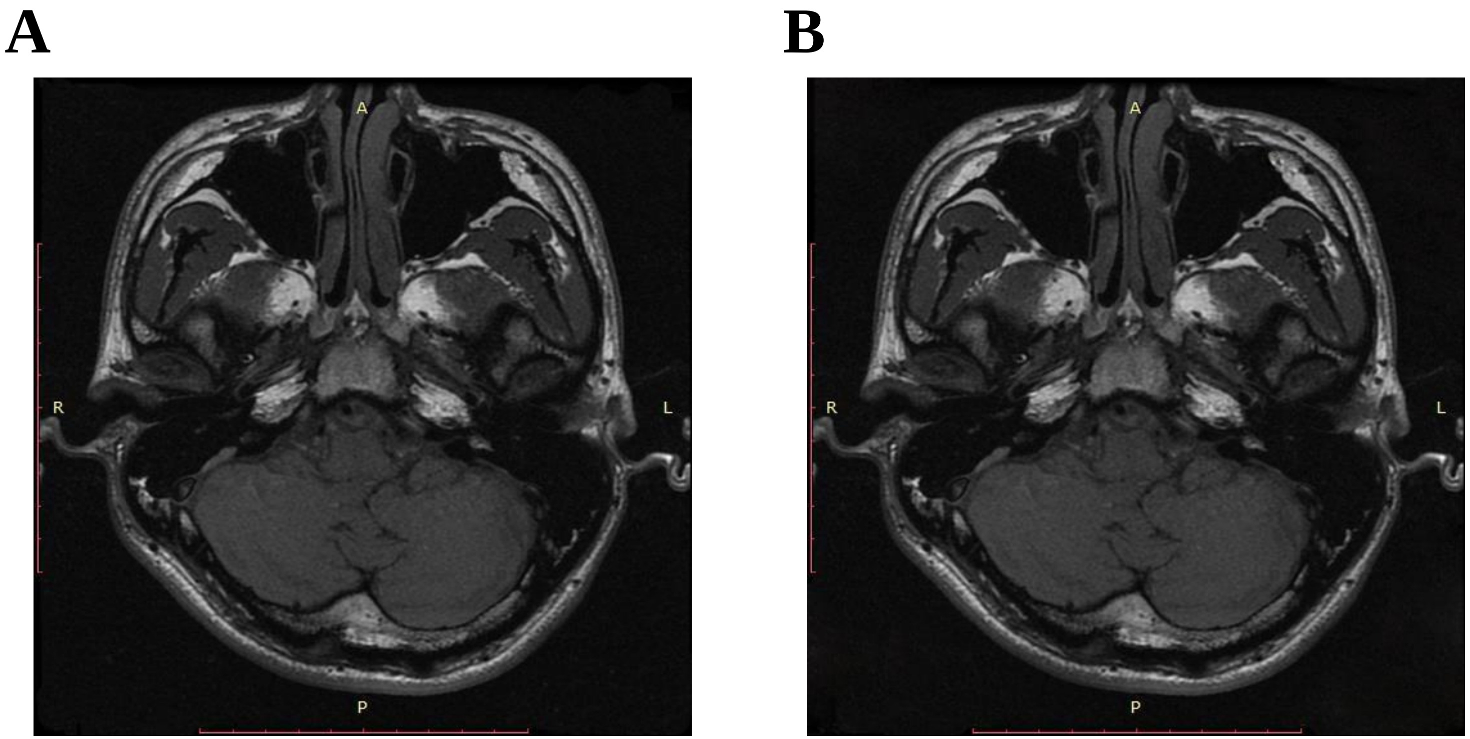

Supplementary figure.3 CT scans showed the EVA in both ears in III-2.(A) the original image of Figure.1G. (B) the original image of Figure.1H.

A **B**

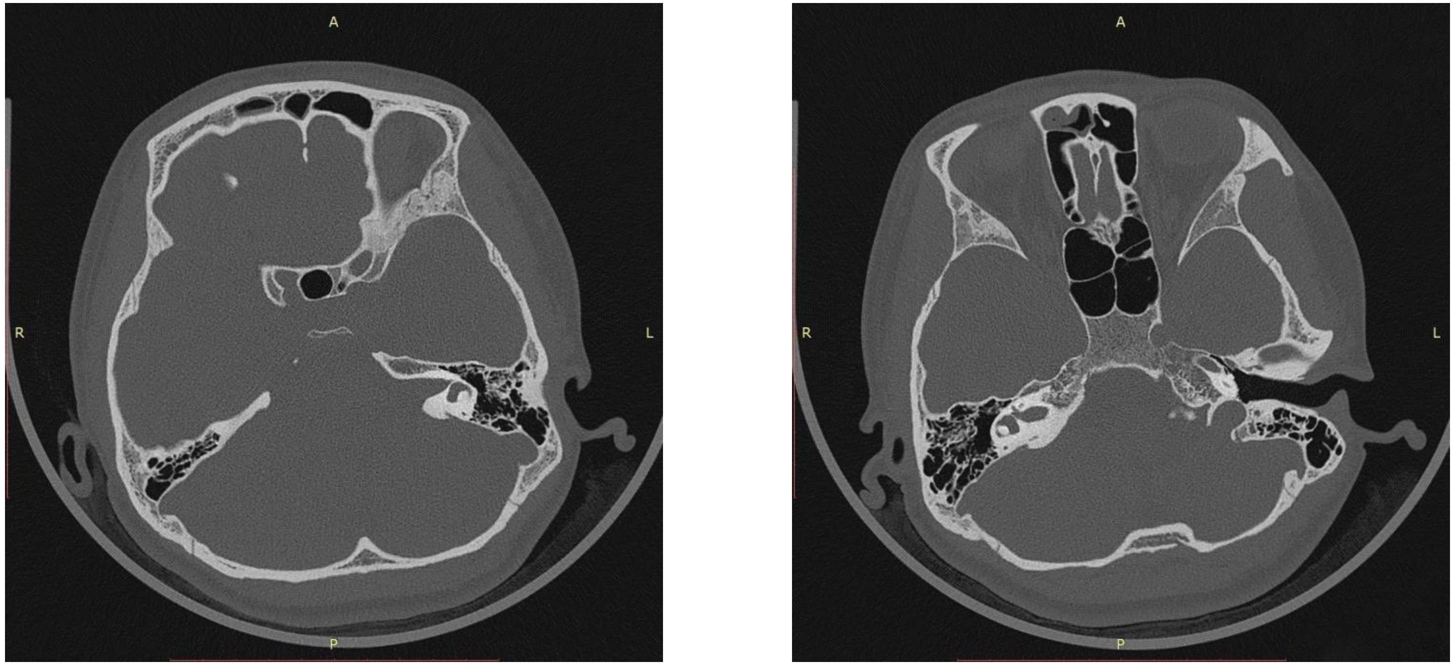

Supplementary figure.4 The original ,unprocessed versions of figure.5B. (A) the original blot of ACTIN. (B) the original blot of SLC26A4.

A

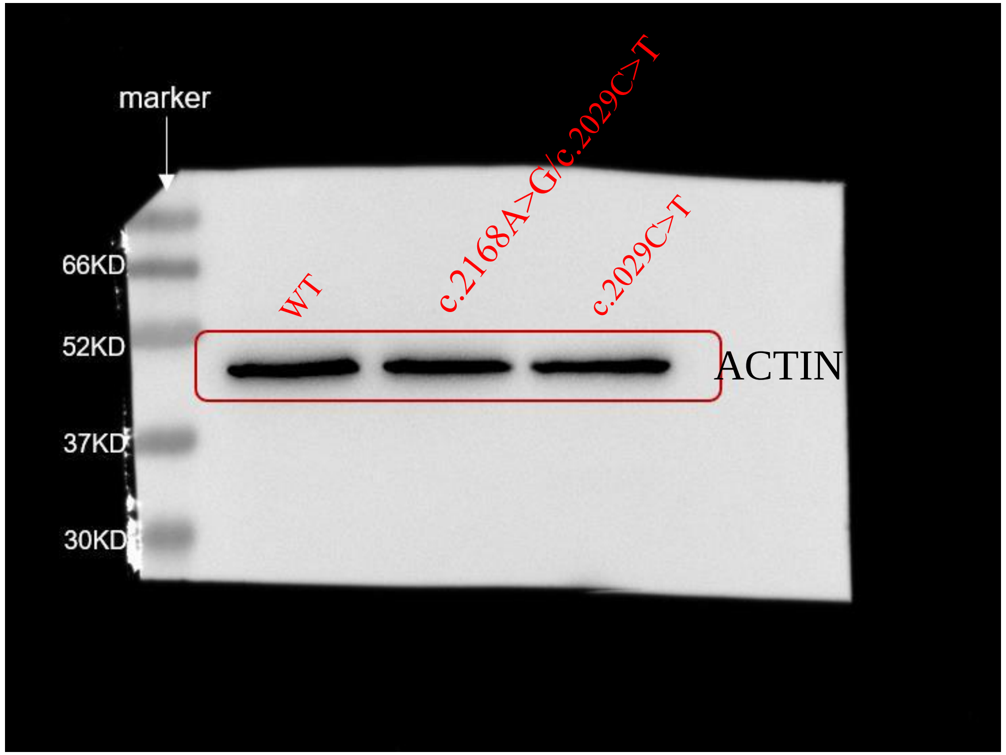

B

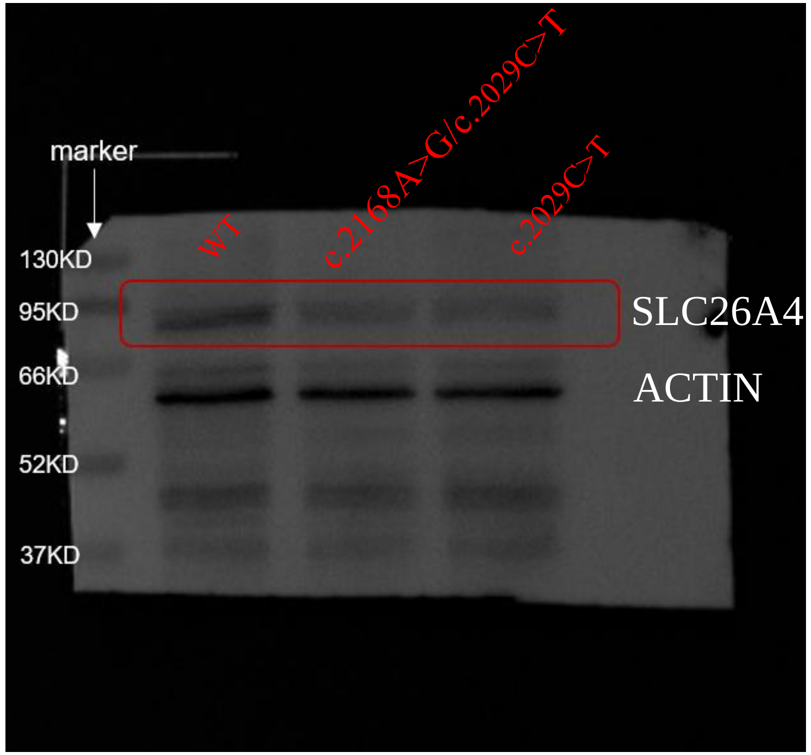

Supplement: Supplementary file 1 — Additional file 1 . The original data of imaging examination and Western bloting examination. [file 12920_2022_1200_MOESM1_ESM.pdf]
